# Implementation of an Outpatient HD-MTX Initiative

**DOI:** 10.3389/fonc.2021.773397

**Published:** 2022-01-20

**Authors:** Kelsey Sokol, Kelley Yuan, Maria Piddoubny, Ellen Sweeney, Anne Delengowski, Katlin Fendler, Gloria Espinosa, Judith Alberto, Patricia Galanis, Carol Gung, Meghan Stokley, Mercy George, Mary Harris, Ubaldo Martinez-Outschoorn, Onder Alpdogan, Pierluigi Porcu, Adam F. Binder

**Affiliations:** ^1^ Department of Medical Oncology, Thomas Jefferson University, Philadelphia, PA, United States; ^2^ Sidney Kimmel Medical College, Philadelphia, PA, United States; ^3^ Department of Pharmacy, Thomas Jefferson University, Philadelphia, PA, United States; ^4^ Department of Nursing, Thomas Jefferson University, Philadelphia, PA, United States; ^5^ Division of Hematologic Malignancies and Hematopoietic Stem Cell Transplant, Department of Medical Oncology, Sidney Kimmel Cancer Center, Thomas Jefferson University, Philadelphia, PA, United States

**Keywords:** health care delivery, diffuse large B cell lymphoma, CNS prophylactic treatment, quality improvement, chemotherapy – oncology

## Abstract

**Introduction:**

Methotrexate (MTX) a folate antagonist is often given in high doses (≥500 mg/m^2^) to treat a variety of disease processes. While inpatient administration has been the norm, outpatient administration, has been shown to be safe, effective, and patient centered. Here in we describe development of an outpatient HDMTX protocol and our initial experience.

**Methods:**

All patients were to receive their first cycle of HDMTX in the hospital to ensure they tolerate it well and also to use this time to assist in training for home administration. The outpatient protocol involved continuous IV sodium bicarbonate, along with oral leucovorin and acetazolamide. Patients were required to visit the infusion center daily for labs and methotrexate levels. Clear criteria for admission were developed in the case of delayed clearance or methotrexate toxicity.

**Results:**

Two patients completed the safety run-in phase. Both patients tolerated treatment well. There were no associated toxicity. Methotrexate cleared within 3 days for all cycles. Both patients were able to follow the preadmission instructions for sodium bicarbonate and acetazolamide. The patients reported adequate teaching on the protocol and were able to maintain frequency of urine dipstick checks.

**Conclusion:**

We developed and implemented an outpatient protocol for high dose methotrexate. This study largely details the development of this protocol and its initial safety evaluation. More work needs to be done to assess its feasibility on a larger number of patients who receive more cycles in the outpatient setting.

## Introduction

Methotrexate (MTX) is a folate antagonist used across a wide range of diseases, with dose levels classified as low or high (1). High-dose methotrexate (HDMTX) consists of doses ≥500 mg/m^2^, used for the treatment of primary and secondary CNS lymphoma (PCNSL), leptomeningeal metastases, and osteosarcoma, as well as central nervous system (CNS) prophylaxis in patients with leukemia and high-risk lymphoma. These doses are potentially lethal without administration of intravenous and/or oral leucovorin to rescue normal cells from apoptosis, specifically in the bone marrow and GI tract (2, 3). In addition to leucovorin, normal renal clearance of MTX is required to quickly eliminate the drug, supported by aggressive hydration and urinary alkalinization. Toxicities often occur as a result of the high dose and duration of exposure, and include renal toxicity, hepatotoxicity, stomatitis, myelosuppression, rash, pneumonitis, and encephalopathy (4). To ensure safe administration, HDMTX is most often administered in the hospital setting with an average length of stay around 4-5 days, barring any treatment complications. For patients receiving HDMTX for Primary CNS lymphoma or CNS prophylaxis for DLBCL, these inpatient hospital admissions, which most commonly are scheduled every 2 weeks, have the potential to negatively impact a patient’s quality of life and contribute to the growing financial burden on the health care system.

There is experience with outpatient administration of HDMTX in the pediatric population. Two small prospective studies, one in pediatric acute lymphoblastic leukemia (ALL) and one in pediatric osteosarcoma, showed the safety and feasibility of this approach in the appropriate patient population, reducing cost and increasing quality of life by allowing patients to remain out of the hospital (5, 6). Two large, retrospective studies in pediatric populations from Memorial Sloan Kettering Cancer Center (MSKCC) and the Children’s Hospital at Montefiore support these findings (7, 8). While NCCN has published a toolkit to implement outpatient HDMTX (9), few studies have been published and none of these include the adult patient population, presenting a significant gap in the literature (10, 11). Studies are needed to examine the feasibility and safety in this population, as comorbidities, organ dysfunction, and social challenges seen in adults differ greatly from the pediatric population (12).

Prior to the development of an outpatient HDMTX protocol in the adult population, we performed a retrospective review of all patients receiving inpatient HDMTX at Thomas Jefferson University, an urban academic tertiary care medical center, between January 1, 2018 and October 31, 2019 to determine the distribution and rate of toxicities in adult patients at our institution. Details of this study have been presented (13). The most common toxicity observed in this population was acute kidney injury (AKI), with an incidence of 21% in patients receiving HDMTX as prophylaxis [median dose 3.5 g/m2 (1-3.5 g/m2)] compared to 33% in patients receiving HDMTX as treatment [median dose 3.5 g/m2 (range 0.25-12 g/m2)] for CNS disease. Additionally, all AKI in the prophylactic group was grade II (>1.5 - 3.0 x baseline; >1.5 - 3.0 x ULN) or lower according to CTCAE version 5.0 and resolved. Other toxicities in patients receiving HDMTX for CNS prophylaxis was minimal. Based on these results, we initiated an outpatient HDMTX program in patients requiring CNS prophylaxis for diffuse large B-cell lymphoma (DLCBL). Herein we present the development of our outpatient HDMTX protocol and results of the safety run-in phase.

## Methods

We created an inter-professional, multi-disciplinary team to develop the outpatient HD-MTX protocol. Our team included an oncologist, oncology fellow, infusion center managers, nurse educators, inpatient and outpatient nursing staff, advanced practice providers, oncology pharmacists and home infusion service nurses and pharmacists. Protocol development followed an iterative process and the final protocol is referenced in **Appendix A**. Once developed, the protocol was implemented and modified through an iterative process with each cycle of HD-MTX administered.

Complete patient eligibility criteria are listed in the protocol, including a diagnosis of DLBCL requiring CNS prophylaxis (as determined by their provider), age 18-70 years, ECOG performance status ≤ 2, normal renal function (CrCl ≥60 mL/min), no history of color blindness (to be able to read the urine dipstick), and adequate social support. Patients who clear their first administration of HDMTX within 3-5 days without complications qualify to receive cycles 2 and 3 in the outpatient setting. Both the patient and caregiver are educated on reading the urine dipsticks. While the optimal method of CNS prophylaxis has yet to be defined by prospective randomized studies, each patient receives a currently accepted standard of 3 cycles of HDMTX at a dose of 3.5 gm/m^2^. HDMTX can be given alternating with R-CHOP after even cycles (i.e. 2, 4, and 6) or at the end of 6 cycles of systemic treatment. All patients complete cycle 1 of the protocol as an inpatient to ensure clearance of the MTX without complications on the outpatient protocol.

During the inpatient stay for cycle 1, nursing and pharmacy education is performed. Patients are taught how to use the urine dipstick for pH monitoring. The frequency of monitoring is discussed and reinforced. Scheduled medications are reviewed. Pharmacists discuss with patients concomitant medications such as sulfonamides, non-steroidal anti-inflammatory drugs, and proton pump inhibitors should be avoided until after methotrexate clearance. The home infusion team visits with the patient, ensures insurance will cover all related costs, reviews the process for delivery of materials and supplies and also provides an emergency number for 24 hour access in case the infusion pump malfunctions or other questions arise. Patients are also encouraged to call the cancer center at any time to speak with a physician if any concerns or questions arise during the home infusion process.

The protocol is initiated as follows. Oral sodium bicarbonate 1950mg every 6 hours and oral acetazolamide 500mg every 6 hours is initiated at home, three and one days prior to MTX infusion, respectively. On treatment day, patients receive HDMTX, given over 4 hours, in the infusion center, and continuous IV fluids with normal saline (later changed to IV sodium bicarbonate allowing discontinuation of oral sodium bicarbonate) through central venous catheter is initiated. No recommendations were made on minimum or maximum daily oral fluid intake as the patient will receive the necessary daily volume of fluids intravenously. Patients monitor urine pH at home *via* urine dipstick every 2 hours on day 1 followed by every 4 hours thereafter while awake. Nightly acetazolamide 500mg is continued, with additional dose every 6 hours as needed for urine pH <7.5. Starting 24 hours after MTX infusion, patients begin oral leucovorin 25 mg every 6 hours. A record is kept of side effects, timing of medications, and urine pH levels (see **Appendix B**). Patients present daily to the infusion center for toxicity check, medication review, and blood draw including complete blood count, comprehensive metabolic panel, and methotrexate levels (starting on day 3, but this was later modified to day 2). Urine pH is checked daily through central laboratory to ensure correlation with urine dipsticks. Oral leucovorin is increased to 25mg every 3 hours as needed based on the nomogram (**Appendix C**). According to our standard of care practice, if the patient fails to show up for a scheduled appointment, they will be called by the care team to investigate further. Indications for admission to the hospital include serum Cr ≥1.5x baseline, toxicity unable to be managed as outpatient, or need to increase leucovorin dose to >25mg every 3 hours (given limit on oral bioavailability) (14). Once methotrexate level is ≤0.1µmol/L, patients are disconnected from IV fluid infusion and stop oral acetazolamide. Oral leucovorin is continued and oral sodium bicarbonate is restarted for 3 additional days.

As part of the implementation process, a safety run-in phase was designed such that the first 5 cycles on the outpatient protocol were administered in the inpatient setting. This run-in was implemented to allow for any necessary changes to the protocol to be made if unexpected barriers arose.

## Results

Two patients completed the safety run-in phase. The first patient, a 46-year-old man with no comorbidities, received all three cycles of HDMTX for CNS prophylaxis as an inpatient on the outpatient protocol. Time to methotrexate clearance was ≤ 2 days for all three cycles. No AKI or other toxicity was observed. During these cycles, the bedside urine dipsticks that were utilized reported urine pH readings 0.5 higher than the lab urine pH. As a result, an alternative urine dipstick test was used for the second patient during the run-in phase.

The second patient, a 28-year-old man with no comorbidities, received cycle 1 and 2 inpatient on the outpatient protocol, with the third cycle completed outpatient. Time to methotrexate clearance was ≤ 3 days for all three cycles. Again, no AKI or other toxicity was observed. With the change in urine dipstick, the lab-based pH values and those obtained at the bedside correlated.

Both patients were able to follow the preadmission instructions for sodium bicarbonate and acetazolamide. The patients reported adequate teaching on the protocol and were able to maintain frequency of urine dipstick checks. Several logistical improvements were made to the protocol based on patient feedback and process review.

## Discussion

Care models for the administration of outpatient chemotherapy are becoming increasingly popular, implemented with the goal to improve patient quality of life and decrease costs to the patient and health care system. While the safety and feasibility of outpatient HDMTX has been published in pediatric populations, and a toolkit has been suggested by NCCN, this protocol is one of the few publication we are aware of detailing implementation of outpatient HDMTX administration in adult patients. Our safety run-in phase demonstrated feasibility and safety of the outpatient protocol for patients with DLBCL requiring CNS prophylaxis, although we acknowledge the small sample size (n=2) and young age of these patients may bias the initial results. As the next phase of this implementation initiative, a prospective observational study is being performed with the primary objective to evaluate the rate of methotrexate toxicity, particularly renal toxicity, in the outpatient setting as compared to the rate of toxicity of our historical control. Secondary objectives include comparing the effects on treatment burden and cost burden for these care models, the total cost and out-of-pocket cost for patients, as well as quality of life of inpatient versus outpatient treatment modalities. This is being assessed through use of the patient reported Treatment Burden Questionnaire (TBQ) (15), the Cost Burden Survey (16), revenue cycle evaluation, and the EORTC-qlq-c30 quality of life survey tool (17).

MSKCC has recently demonstrated feasibility of an outpatient HDMTX protocol integrating glucarpidase into the treatment of primary CNS lymphoma (18). Glucarpidase, a carboxypeptidase enzyme, rapidly inactivates methotrexate to reduce toxicity without crossing the blood brain barrier. Use of glucarpidase for this patient population in the outpatient setting is appropriate given the high risk of AKI and other toxicity seen in our retrospective analysis. However, patients receiving HD-MTX for CNS prophylaxis are a different patient population with lower risk for adverse events including acute kidney injury and thus may not need glucarpidase to effectively receive treatment as an outpatient as has been described in these studies (10, 11). The use of glucarpidase is often restricted to patients who meet specific criteria due to its high cost. Our protocol aims to provide a patient centered approach while at the same time limits cost and financial burden of this outpatient regimen. We recognize that our protocol requires significant effort and commitment on behalf of the patient and their family, and is therefore limited to a subset of patients with adequate social support and transportation resources. Additionally, the success of outpatient chemotherapy care models requires a supportive, multidisciplinary team.

## Data Availability Statement

The raw data supporting the conclusions of this article will be made available by the authors, without undue reservation.

## Author Contributions

All authors listed have made a substantial, direct, and intellectual contribution to the work, and approved it for publication.

## Conflict of Interest

The authors declare that the research was conducted in the absence of any commercial or financial relationships that could be construed as a potential conflict of interest.

## Publisher’s Note

All claims expressed in this article are solely those of the authors and do not necessarily represent those of their affiliated organizations, or those of the publisher, the editors and the reviewers. Any product that may be evaluated in this article, or claim that may be made by its manufacturer, is not guaranteed or endorsed by the publisher.
