# Supplementary material for: Implementation of an Outpatient HD-MTX Initiative
Source: Front Oncol. 2022 Jan 20;11:773397. doi: 10.3389/fonc.2021.773397 (PMC8811119; doi:10.3389/fonc.2021.773397)
Supplement: Supplementary file 1 [file Table_1.docx]

**Supplementary Materials**

**APPENDIX A**

**High Dose Methotrexate: SOP for Outpatient Administration**

**Purpose**

While inpatient chemotherapy allows close patient monitoring during treatment, outpatient chemotherapy plans allow safe and easy drug administration, can aid in controlling treatments costs, and most importantly, keeps patients at home. This document serves to standardize the practice of administering High Dose Methotrexate in the outpatient setting, while maintaining high levels of patient safety and monitoring.

**Beacon Plan Name**

IP/OP Lymphoma High Dose Methotrexate (3.5 gm/m2)

**Overview**

The intent is to treat each patient with 3 cycles of High Dose Methotrexate (3.5 gm/m2) as standard CNS Prophylaxis. As a safety precaution, each patient will receive Cycle 1 in the inpatient setting. Provided they clear their methotrexate in a safe and timely manner on an oral regimen, they will be candidates to receive Cycles 2-3 in the outpatient setting.

**Inclusion Criteria**

- Diagnosis of systemic DLBCL requiring CNS prophylaxis
- Males and females between age 18-70
- ECOG PS ≤2
- CrCl ≥60 mL/min
- Ability to come to the office on a daily basis in the morning for labs and symptoms check
- Ability to read Urine pH strips
- Support system in place to come to the office or ED at any time of day if required by care team
- Insurance plan allows labs to be drawn at Center City 925 Chestnut location
- Able to pay out of pocket costs for oral supportive medications.
- Has PICC/Midline or PORT for reliable IV Fluid administration.

**Exclusion Criteria**

- Inability to comply with independent monitoring and medication dosing or inadequate social support, as determined by treating physician
- Unable to have labs drawn at Center City 925 Chestnut location (e.g. capped to LabCorp)
- Unable to pay out of pocket costs of oral medications
- Unable to read Urine pH strips
- Age >70
- ECOG PS >2
- CrCl <60 mL/min
- Primary CNS Lymphoma
- Lack of appropriate IV access

**Access Required**

PICC/Midline or port

**Home Medications/Prescriptions**

1. ondansetron (ZOFRAN) 8 mg tablet, Disp-30 tablet, R-2
   - Take 1 tablet every 8 hours PRN by mouth for nausea/vomiting
2. prochlorperazine (COMPAZINE) 10 mg tablet, Disp-60 tablet, R-2
   - Take 1 tablet every 6 hours PRN by mouth for nausea/vomiting
3. sodium bicarbonate 650 mg tablet, Disp-75 ~~120~~ tablet, R-2
   - Take 3 tablets (1,950 mg) every 6 hours by mouth, begin the morning 3 days prior to methotrexate administration up until methotrexate is administered, then hold while receiving intravenous sodium bicarbonate. Restart as directed by your care team once intravenous sodium bicarbonate is completed, take 3 tablets (1,950 mg) by mouth every 6 hours for 3 additional days
4. acetazolamide (DIAMOX) 250 mg tablet, Disp-80 tablet, R-2
   - Take 2 tablets (500 mg) every 6 hours the day prior to methotrexate administration.
   - After methotrexate administration take 2 tablets (500 mg) every 6 hours; hold if urine pH > 7.5. Take 2 tablets (500 mg) every night at bedtime regardless of urine pH.
5. leucovorin (WELLCOVORIN) 25 mg tablet, Disp-52 ~~48~~ tablet, R-2
   - Take 1 tablet every 6 hours by mouth starting 24 hours following the beginning of your methotrexate infusion, increase frequency as directed
6. multiple urine test strips, Disp-100 strip, R-1 (Provided by Dr. Binder)
   - Check urine every 2 hours on day 1, then every 4 hours on subsequent days

**Infusion Center Accommodations**

Cycle 2-3, Day 1

Treatment day: Monday

Chair Time: 270 minutes

**Treatment Team Visits**

All cycles, once Day -4 to -3

Cycle 2-3, Day 2-X

**Schedule (Cycle 1 to be administered inpatient, Cycle 2-3 to be administered outpatient)**

**Cycle 1:**

Day -4 to -3

- Visit by treatment team
- Confirm no evidence of volume overload, pleural effusions or ascites
- Confirm HDMTX order set has been signed
- Patient to bring prescription for oral acetazolamide, sodium bicarbonate, and leucovorin to visit for review
- Care team to provide urine dipsticks
- Patient education via care team:
  - Medication Administration
  - Urine Dipstick and pH Log
  - IV sodium bicarbonate fluid bag exchange

Day -2

- Patient to start sodium bicarbonate 1950mg PO every 6 hours

Day 0

- Patient to take acetazolamide 500mg PO every 6 hours

**Cycle 1 (Inpatient):**

Day 1:

- Prior to admission
  - Take acetazolamide 500mg PO once in AM prior
  - Take sodium bicarbonate 1950mg PO once in AM prior
- Admission to hospital
  - Baseline admission labs
  - Check urine pH via lab and via patient’s dipstick to confirm correlation, goal > 7.5
  - HDMTX administration
  - IV sodium bicarbonate initiated via continuous infusion pump at 125cc/hr
  - Check urine pH via patient’s dipstick and lab every 2 hours
    - Urine pH does not need to be checked while patient is sleeping, however, can be checked if patient awakes to void at appropriate interval
  - Scheduled medications:
    - Acetazolamide 500mg PO nightly
    - Acetazolamide 500mg PO every 6 hours, hold if urine pH > 7.5
    - Sodium bicarbonate PO on HOLD until directed to restart by care team

Day 2-X

- Continue IV sodium bicarbonate via continuous infusion at 125cc/hr
- Check urine pH via lab and patient’s dipstick every 4 hours
  - Does not need to be checked while patient is sleeping, however, can be checked if patient awakes to void at appropriate in interval
- Scheduled medications:
  - Acetazolamide 500mg PO nightly
  - Acetazolamide 500mg every 6 hours, hold if urine pH > 7.5
  - Leucovorin 25mg PO every 6 hours starting 24 hours after beginning of MTX infusion
    - Increase leucovorin frequency per nomogram
    - Max oral dose is 25mg every 3 hrs

Day X: MTX level ≤0.1 μmol/L

- Stop IV sodium bicarbonate
- Stop acetazolamide
- JHIS consulted for coordination for future outpatient cycles
- Discharge home
- Take oral sodium bicarbonate and leucovorin x 3 more days

**Cycle 2-3 (Outpatient)**

**Days -4 to 0 (see above)**

Day 1:

- At home
  - Take sodium bicarbonate 1950mg PO once in AM prior
  - Take acetazolamide 500mg PO once in AM prior
  - Check urine dipstick prior to arriving in the OIC and notify care team if pH not >7.5
- Present to OIC: 2^nd^ Floor
  - Baseline labs
  - Check urine pH via lab and via patient’s dipstick to confirm correlation, goal >7.5
  - HDMTX administration in OIC
  - JHIS called to connect patient to IV sodium bicarbonate
  - Nurse to fill out patient’s paperwork with methotrexate infusion start time and oral leucovorin start time
- Scheduled medications at OIC:
  - Acetazolamide 500mg PO once, hold if urine pH > 7.5
  - Sodium bicarbonate 1950mg PO once, hold if urine pH > 7.5
- Scheduled medications:
  - Acetazolamide 500mg PO nightly
  - Acetazolamide 500mg PO every 6 hours, hold if urine pH > 7.5

Day 2-X:

- Scheduled medications:
  - Acetazolamide 500mg PO nightly
  - Acetazolamide 500mg PO every 6 hours, hold if urine pH > 7.5
  - Leucovorin 25mg PO every 6 hours starting 24 hours after beginning of MTX infusion
- Morning appointment in OIC with care team: 4^th^ Floor
- Check labs (CBC, CMP, urine pH)
  - MTX levels will be checked Day 3-X
- Toxicity check, review medications and compliance, review urine pH log
- Adjust leucovorin frequency to 25mg every 3 hours if needed based on methotrexate level and nomogram
  - If higher dose needed, admit to hospital and convert to IV leucovorin
- Admit to hospital if
  - - - Serum Cr ≥1.5x baseline
      - Other toxicity unable to be managed as outpatient (i.e. nausea or vomiting, not controlled with oral anti-emetics,
      - Leucovorin dose >25mg every 3 hours is needed

Day X: MTX level ≤0.1µmol/L

- JHIS to discontinue IV sodium bicarbonate
- Stop acetazolamide
- Continue PO leucovorin for 3 more days
- Restart PO sodium bicarbonate for 3 more days

**APPENDIX B**

| HOME TRACKING SHEET | | |
| --- | --- | --- |
| Urine pH (Time/pH level) | Acetazolamide Dose (Time/Dose) | Side Effects |
|  |  |  |
|  |  |  |
|  |  |  |
|  |  |  |
|  |  |  |
|  |  |  |
|  |  |  |
|  |  |  |
|  |  |  |
|  |  |  |
|  |  |  |
|  |  |  |
|  |  |  |
|  |  |  |
|  |  |  |
|  |  |  |
|  |  |  |
|  |  |  |
|  |  |  |
|  |  |  |
|  |  |  |
|  |  |  |
|  |  |  |
|  |  |  |
|  |  |  |
|  |  |  |
|  |  |  |
|  |  |  |
|  |  |  |
|  |  |  |
|  |  |  |
|  |  |  |
|  |  |  |
|  |  |  |
|  |  |  |
|  |  |  |
|  |  |  |
|  |  |  |
|  |  |  |
|  |  |  |
|  |  |  |

**APPENDIX C**

**Leucovorin Rescue Dosing**

| **Methotrexate Elimination** | | | | |
| --- | --- | --- | --- | --- |
|  | **Normal MTX Elimination** | **Slow MTX Elimination** | **Delayed MTX Elimination** | **AKI/MTX Toxicity** |
| **Time (hr)** | **MTX Level (µM)** | **MTX Level (µM)** | **MTX Level (µM)** | **MTX Level (µM)** |
| 24 | <10 | 10-25 | 25-50 | ≥50 or 100% increase in sCr |
| 48 | <1 | 1-5 |  | ≥5 or 100% increase in sCr |
| 72 | <0.2 |  |  |  |
| Leucovorin Dose | 25mg IV/PO Q6H x 10 doses | 25mg IV/PO Q3H until MTX <0.05 µM (If at home Q6H if sleeping) | 100mg IV Q3H until MTX <0.05 µM | 250mg IV Q3H hours until MTX <1 µM, and then 25mg IV/PO Q3H until MTX <0.05 µM  *Discussion about need for flat dose glucarpidase (1,000 units x1) |
| **All increased doses should be carried forward to future treatment cycles. Pharmacists may adjust the leucovorin dose, but not dosing frequency, to reflect this increase, per protocol. | | | | |
